# Advances in targeted delivery of mRNA into immune cells for enhanced cancer therapy

**DOI:** 10.7150/thno.93745

**Published:** 2024-09-03

**Authors:** Linzhuo Huang, Zhiquan Huang, Yuxuan Zhang, Chunhao Lin, Zixuan Zhao, Rong Li, Phei Er Saw, Xiaoding Xu

**Affiliations:** 1 Guangdong Provincial Key Laboratory of Malignant Tumor Epigenetics and Gene Regulation, Medical Research Center, Sun Yat-Sen Memorial Hospital, Sun Yat-Sen University, Guangzhou 510120, P. R. China.; 2 Guangzhou Key Laboratory of Medical Nanomaterials, Sun Yat-Sen Memorial Hospital, Sun Yat-Sen University, Guangzhou 510120, P. R. China.; 3 Nanhai Translational Innovation Center of Precision Immunology, Sun Yat-Sen Memorial Hospital, Foshan 528200, P. R. China.; 4 The Second Affiliated Hospital, Hengyang Medical School, University of South China, Hengyang 421001, P. R. China.

**Keywords:** Messenger RNA (mRNA), immune cells, nanoparticles (NPs), targeted delivery, cancer immunotherapy

## Abstract

Messenger RNA (mRNA) therapy has been applied to the treatment of various human diseases including malignant tumors. Increasing evidences have shown that mRNA can enhance the efficacy of cancer immunotherapy by modulating the functions of immune cells and stimulating their activity. However, mRNA is a type of negatively charged biomacromolecules that are susceptible to serum nucleases and cannot readily cross the cell membrane. In the past few decades, various nanoparticles (NPs)-based delivery systems have been rationally designed and developed to facilitate the intracellular uptake and cytosolic delivery of mRNA. More importantly, by means of the specific recognition between the targeting ligands decorated on NP surface and receptors specifically expressed on immune cells, these mRNA delivery systems could be functionalized to target immune cells to further enhance the mRNA-based cancer immunotherapy. In this review, we briefly introduced the advancements of mRNA in cancer therapy, discussed the challenges faced by mRNA delivery, and systematically summarized the recent development in NPs-based mRNA delivery systems targeting various types of immune cells for cancer immunotherapy. The future development of NPs-mediated targeted mRNA delivery and their challenges in clinical translation are also discussed.

## Introduction

Messenger RNA (mRNA) is a single-stranded ribonucleic acid transcribed from DNA that has the capability to encode nearly all proteins. Unlike siRNA or CRISPR interference, which operate on the principle of reducing intracellular gene expression, mRNA is primarily used to compensate for genetic mutations or deficiencies, thereby elevating the levels of specific proteins 1. Increasing research demonstrates significant advantages of mRNA therapy. In comparison to DNA-based therapies, mRNA can exert its effects without needing to enter the cell nucleus, and it carries a relatively high transfection efficiency and safety due to the absence of the risk of random insertional mutations 2. Additionally, in contrast to transient protein or peptide drug therapies, mRNA allows for sustained translation, resulting in a prolonged therapeutic effect 3. Therefore, over the past few decades, mRNA has been used in various biological applications, including protein replacement therapy, tissue engineering, gene editing, cancer vaccines, *etc* 4. We briefly summarize the applications of mRNA beyond immunotherapy (Table 1), and the application in cancer immunotherapy will be described in detail in the next section. In the field of cancer immunotherapy, mRNA can complement immune-related deficient proteins, modulate the immune system, and improve the efficacy of cancer immunotherapy. What is even more crucial is that, in immunotherapy, each type of immune cell requires specific mRNA to carry out its unique and vital immune functions 5. Therefore, it is essential to deliver mRNA to specific subsets of immune cells.

To function effectively, mRNA needs to be delivered to cellular compartments. However, mRNA is sensitive to enzymatic degradation and faces challenges entering the cell cytoplasm 11. To overcome these obstacles, mRNA molecules can be encapsulated in nanoparticle carriers. In the field of immunotherapy, to achieve optimal therapeutic effects and minimize side effects, it is crucial to deliver mRNA to specific subsets of immune cells, ensuring the uptake of genetic material and the expression of proteins 5. For instance, delivering mRNA to dendritic cells (DCs) enhances antigen presentation capabilities, thereby improving anti-tumor immune responses. Delivering mRNA to T and natural killer (NK) cells enhances their cytotoxic effects; Delivering mRNA to tumor-associated macrophages (TAMs) reverses immune suppression, and delivering mRNA to B cells enhances adaptive immune functions, *etc* 12. Therefore, research on immune cell-targeted delivery based on mRNA therapy is crucial, as it will contribute to advancing precision medicine. Surface modification of nanoparticles or the selection of nanoparticles with specific targeting functions can enhance the immune cells targeting, enabling the delivery of higher concentrations of mRNA to specific immune cell populations. Optimizing these delivery systems will drive progress in mRNA-based immunotherapy. In this review, we first introduced the progress of mRNA in cancer immunology, providing a brief overview of the challenges faced by mRNA delivery and existing delivery systems. Subsequently, we described the advancements in immune cell targeting methods based on these cancer immunotherapy targets and mRNA delivery systems. Finally, we summarized the potential strategies for mRNA targeted delivery and discussed the prospects and challenges of designing nanoparticles to enhance targeted immunotherapy.

## The progress of mRNA therapy in cancer immunology

**Figure 1:**
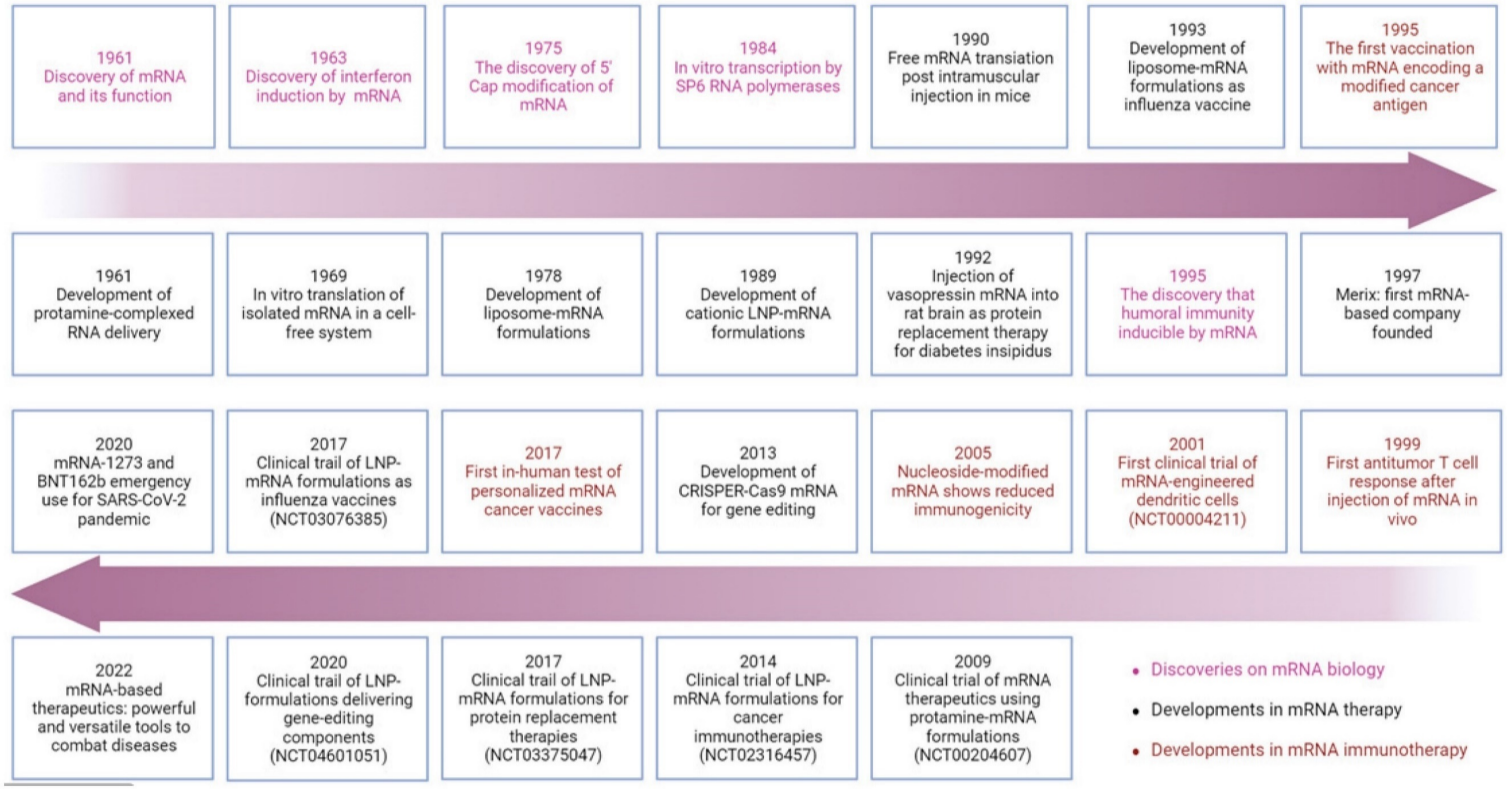
The history of important discoveries in mRNA biology and developments in mRNA therapy and immunotherapy

Cancer immunotherapy is a treatment strategy based on the ability of the immune system to recognize and eliminate cancer cells. The success of a series of clinical drugs targeting cancer immunotherapy demonstrates the potential of the human immune system to fight cancer. Tisagenlecleucel is an autologous CD19-targeted CAR T cell recently approved by the U.S. Food and Drug Administration (FDA), the European Union (EU) and Japan for pediatric or young adult patients with B-ALL 13; Aldesleukin, a recombinant form of human IL-2, was first approved by the FDA for the treatment of metastatic renal cell carcinoma (RCC) in 1992, and subsequently for the treatment of melanoma in 1998 14; Imiquimod (formulated as a 5% topical cream) is widely used in dermatology for the treatment of condyloma acuminatum and may be used to relieve patients with superficial basal cell carcinoma (BCC) 15. This approach was approved by the FDA in 2004. Immune checkpoint blockade (ICB) antibodies have revolutionized cancer treatment, significantly reducing the tumor burden in difficult-to-treat cancer patients 16. However, the success of ICB in solid tumors is limited, with only 13% of patients responding to various cancer types 17. Additionally, most responsive patients eventually experience relapse due to immunotherapy resistance 18. Therefore, there is an urgent need to improve the response rate of immunotherapy for all cancer types. mRNA can be employed in cancer immunotherapy through various treatment modalities, including cancer vaccines, adoptive T-cell therapy, therapeutic antibodies, and immunomodulatory proteins, to mobilize tumor-specific anti-tumor immune responses (Table 2) 19. In recent years, with the development of biotechnology and molecular medicine, mRNA has shown tremendous potential in cancer immunotherapy, as evidenced by recent preclinical and clinical results (Figure 1).

***Cancer vaccines*** have both preventive and therapeutic potential, stimulating and enhancing existing immune responses against tumor antigens. The first mRNA cancer vaccine designed in 1995 demonstrated successful induction of humoral immune responses against encoded tumor-associated antigens (TAAs) in mice 20. In 1999, Zhou and colleagues reported the vaccination using mRNA targeting the tumor-specific antigen gp100 to induce T-cell responses 21. The first clinical trial of prostate-specific antigen (PSA) RNA-loaded DCs vaccine (NCT00004211) began in 2001 22. However, these initial attempts did not significantly propel the widespread application of mRNA therapy in clinical experiments. It wasn't until 2005 that Karikó and Weissman, first reported that mRNA synthesized with pseudouridine instead of uridine could greatly reduce immunogenicity, thereby avoiding recognition and clearance by the immune system 23. This groundbreaking work led them to receive the Nobel Prize in 2023. Subsequently, an increasing number of clinical trials of mRNA vaccines are underway, such as NCT00204607 and NCT02316457^24^. It is noteworthy that scientists reported the first application of personalized mRNA vaccines in humans in 2017 for the treatment of melanoma 25. This study indicated that leveraging individual mutations could pave the way for personalized immunotherapy for cancer patients.

***Adoptive T-cell therapy***, based on chimeric antigen receptor (CAR) in cancer treatment, has been effectively applied in the treatment of liquid tumors but faces challenges in solid tumors. By surface conjugation, specific antibodies target immune cells to deliver mRNA, thereby reprogramming T cells, enabling the possibility of CAR expression *in vivo*.

***mRNA-encoded antibodies***, such as bispecific T-cell engagers (BiTE), bridge the gap between tumor cells and T cells, inducing target-dependent T-cell activation. For instance, BiTE encoding mRNA with 1-methylpseudouridine (RiboMAB) simultaneously targets CD3 and one of three TAAs: claudin-6 (CLDN6), claudin 18.2 (CLDN18.2), or epithelial cell adhesion molecule (EpCAM), which has been shown to induce effective T-cell activation and targeted cancer cell lysis at low concentrations 26.

***mRNA-encoded immunomodulatory proteins*,** including cytokines, toll-like receptors (TLR), chemokines, and costimulatory ligands, can reprogram the tumor immune microenvironment. Hotz* et al.* investigated the anti-tumor effects of intratumoral injection of mRNA encoding four tumor-regressing cytokines (IL-12 single chain, IFN-α, GM-CSF, and IL-15) 27.

## Challenges in mRNA therapy

Despite the significant role of mRNA therapy in cancer immunotherapy, its application is constrained. The major challenges of naked mRNA-based therapy are its biological properties and physical properties, including instability, immunogenicity, large size and negative charge.

### Biological properties

***mRNA instability.*** As there is a large amount of RNA enzyme existence in the cell, the mRNA is easily degraded 32. In addition, the mRNA's secondary structure dsRNA antigen can activate Oligoadenylate synthetases (OASs, the members of IFN-stimulating genes) to produce oligopolyamine to induce Ribonuclease L (RNase-L) to degrade mRNA 33. A variety of chemical modifications can be performed on the mRNA skeleton structure to improve the stability of mRNA, such as modifying the 5' cap structure, optimizing the 5' UTR sequence and adding an appropriate length of poly (A) tail 34, 35.

***mRNA Immunogenicity.*** When mRNA enters the endosome or cytoplasm, the antigen is detected by pattern recognition receptors (PRRs), including TLR3, TLR7, TLR8, and retinoic acid-inducible gene I (RIG-I)-like receptors (RLRs), and these receptors recognize mRNA and stimulate downstream pathways to produce type I interferon and pro-inflammatory cell factors 36. Reducing the immunogenicity of mRNA is essential for enhancing its therapeutic and vaccine applications. Several strategies can be employed for this purpose. One approach is to carefully select nucleotide sequences with lower CpG content, as CpG-rich regions can trigger immune responses 37. Additionally, incorporating chemically modified nucleotides, such as pseudo-uridine and 5-methylcytidine, can minimize the immunogenic potential of mRNA 38. Avoiding the formation of stable secondary RNA structures is also crucial, as these structures may resemble viral RNA and trigger immune responses 39. Optimizing the selection of promoter and terminator sequences can further reduce the immunogenicity of mRNA 40. By employing these approaches and carefully designing mRNA sequences, the risk of immune recognition can be lowered, enhancing the safety and efficacy of mRNA-based therapies and vaccines 41.

### Physical properties

Efficient* in vitro* and* in vivo* delivery of mRNA requires overcoming various barriers. The size of mRNA (300-5,000 kDa, 1-15 kb) is significantly larger than that of siRNA and miRNA analogs (13-15 kDa), antisense oligonucleotides (4-10 kDa) 42. Moreover, mRNA is a negatively charged single-stranded polynucleotide, and it is difficult for naked mRNA to pass through the negatively charged cell membrane.

## Delivery strategies for mRNA therapy

**Figure 2:**
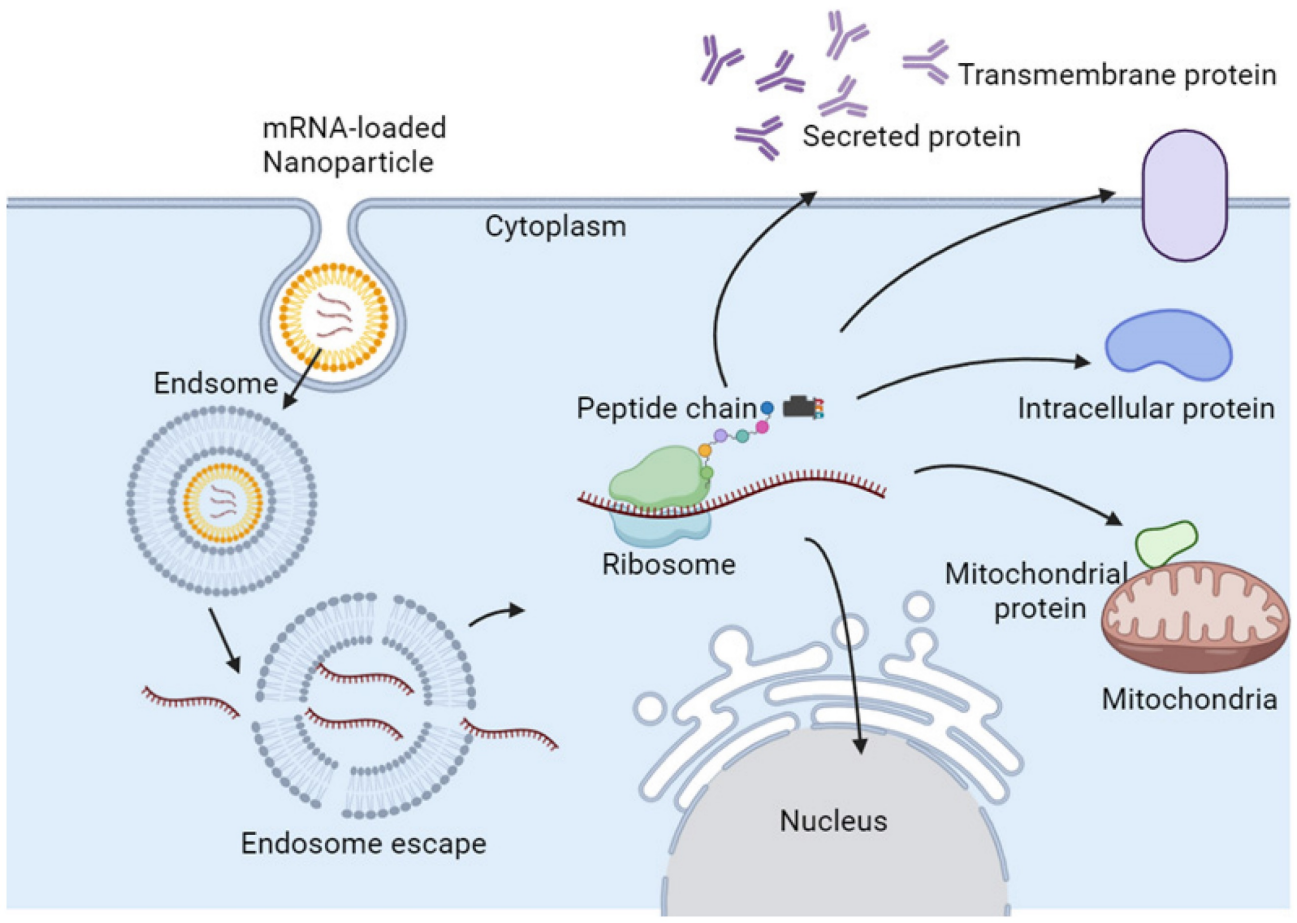
Proposed mechanism of endosomal escape and action of mRNA NPs.

In the case of direct injection of mRNA, only 0.01% of the mRNA can enter the target cell, and most of the mRNA is trapped in the endosome of the target cell and subsequently degraded 43. Eventually, only a few mRNAs escape from the endosome and reach the ribosome for protein translation. Therapeutic mRNAs require more efficient and safer delivery methods, which are critical for enabling promising transformational therapies 44. Therefore, suitable mRNA nanoparticles are necessary for efficient mRNA delivery into most types of cells. Typically, mRNA nanoparticles are ingested by endocytosis, and then mRNA is released from endosomes, where lysosomes will initiate translation and produce any type of protein, including secreted, transmembrane, intracellular, and intramitochondrial proteins (Figure 2) 45.

**Figure 3:**
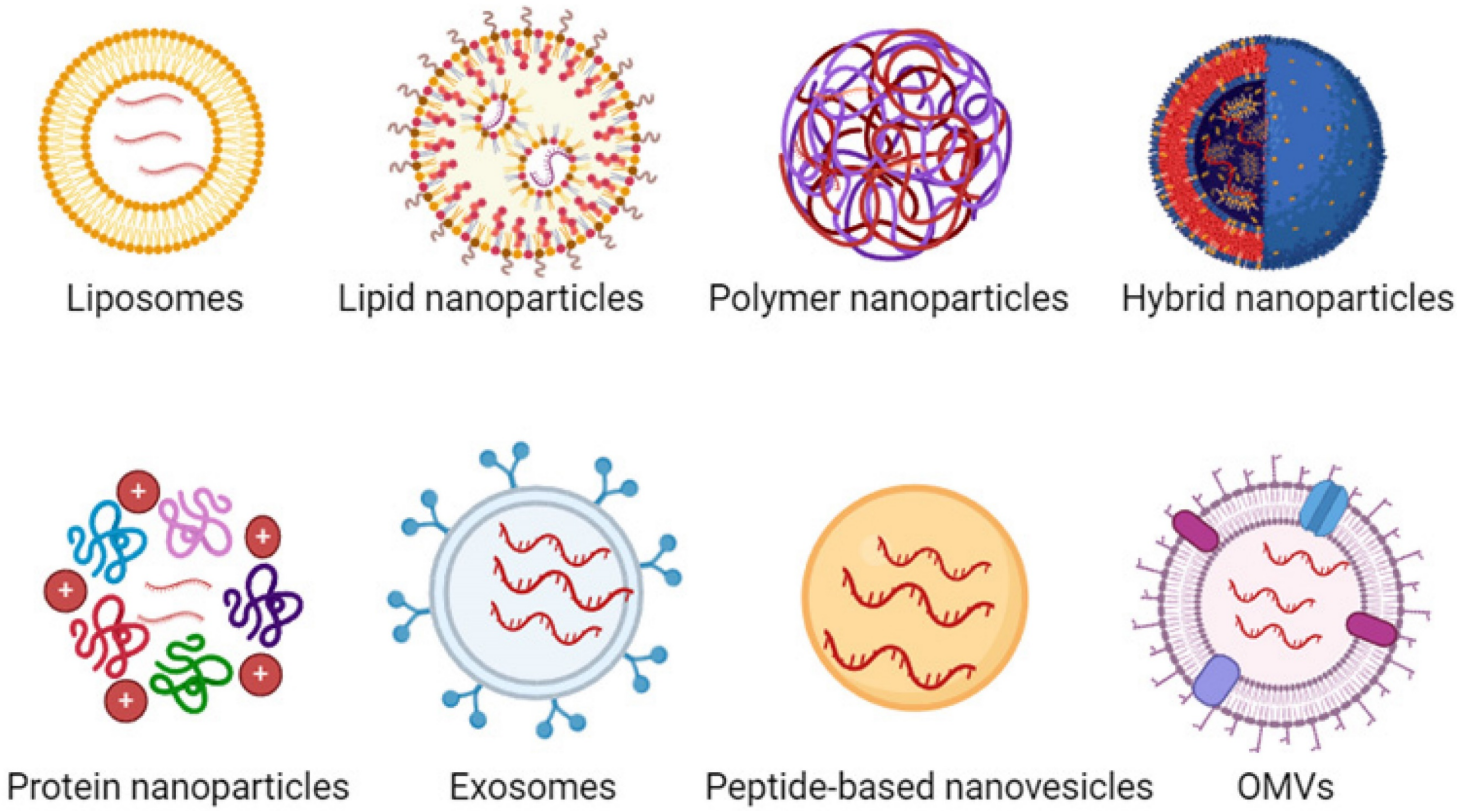
Various types of NPs used for mRNA delivery, including liposomes, LNPs, polymer NPs, lipid-polymer hybrid NPs, protein NPs, exosomes, peptide-based nanovesicles, outer membrane vesicles (OMVs).

In recent years, various nanoparticles (NPs) have been developed for *in vivo* mRNA delivery, including both viral and non-viral carriers. While viral carriers can achieve high levels of transfection in the host, they come with potential immunogenicity and toxicity issues. Therefore, there is an urgent need for non-viral carriers with low immunogenicity and high safety for mRNA delivery. Currently, a variety of non-viral carriers are used for mRNA delivery, such as liposomes, lipid nanoparticles (LNPs), polymer nanoparticles, lipid-polymer hybrid nanoparticles, protein nanoparticles, exosomes, peptide-based nanovesicles, outer membrane vesicles (OMVs) (Figure 3). In addition, we have included table 3 representing the pros and cons of each mRNA NPs.

To gain comprehensive views of various drug delivery platforms for mRNA therapy, readers should read several of the more comprehensive reviews previously published on the topic of mRNA nanodrugs 46. Researchers summarize the types of delivery vectors for mRNA vaccines 47, mRNA delivery vectors for cancer therapy 48-50, types of lipid-associated mRNA delivery vectors 51. LNPs are currently the most intensively studied and clinically advanced mRNA delivery vehicles 52. Among them, cationic and ionizable LNPs are widely used. LNPs typically consist of cationic or ionizable lipids, cholesterol, auxiliary lipids, and polyethylene glycol (PEG)-modified lipids 53. The negatively charged phosphate backbone of mRNA molecules can be efficiently attracted to the positively charged headgroups of cationic lipids through electrostatic interactions, thereby enhancing the encapsulation efficiency of mRNA 54. In clinical research, LNPs have become the state-of-the-art approach for synthetic RNA therapy targeting a range of diseases, such as Patisiran, BNT162b2, and mRNA-1273 55, 56.

Most importantly, owing to the significance of immunotherapy, numerous NPs have been designed and developed for mRNA-based cancer immunotherapy. This mRNA-based cancer immunotherapy includes indirect therapy via delivering mRNA into tumor cells and direct therapy via delivering mRNA into immune cells 57. In this review, we focus on the researches of targeted delivery of mRNA into immune cells for cancer therapy.

## mRNA NPs targeting immune cells

Targeted administration of agents to specific cell subpopulations allows therapeutic agents to concentrate their effects on the target cells, thereby enhancing the therapeutic effect. In addition, immune cells themselves exert their anti-tumor functions through specific mechanisms of action. Therefore, targeted delivery of mRNA to specific immune cells has important application prospects. Specifically, DCs has the key function of initiating T-cell immunity, and DCs can be effectively activated and enhance immunotherapy by tumor-specific antigen (TSA), tumor-associated antigen (TAA), and immune adjuvants (TLR agonists, STING agonists, and C-type lectin receptor (CLR) agonists) 58-61; Co-stimulatory factors OX40 and 4-1BB mRNA can stimulate the proliferation and expansion of CD8^+^ T cells and enhances T cell-mediated anti-tumor immune responses 62; NK cells also have unique stimulatory receptors (NKG2D and NKp46) to promote NK cells activation and killing of tumor cells 63. In this section, we will elaborate on the design strategies for delivering mRNA NPs to specific immune cells for the purpose of precision immune cell targeting. Therefore, we comprehensively summarize the specific information on delivering mRNA to different cell types, including animal models, administration routes, injection routes, dosing amount, dosing times, final results (Table 4).

### mRNA NPs targeting DCs

**Figure 4:**
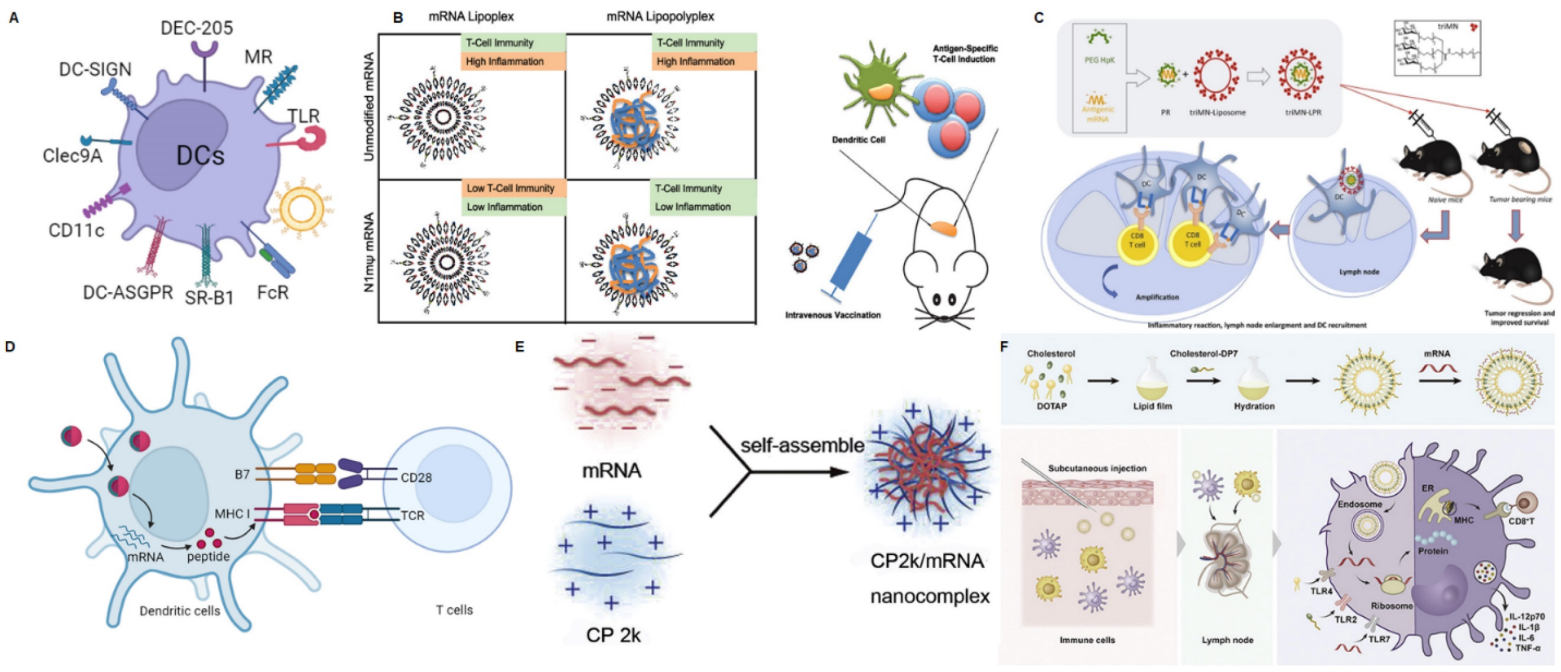
mRNA nanoparticles targeting DCs. (A) Schematic illustration of various receptors expressed on DCs that could be employed to design DCs-targeted NP-mediated delivery systems; (B) Schematic diagram of DCs targeting mRNA lipopolyplexes to exert anti-tumor immunity. Adapted with permission from 64, copyright 2018 ACS Publications; (C) Schematic diagram of DCs targeting antigen-encoding mRNA-loaded triMN-LPR as an anti-tumor vaccine formulation. Adapted with permission from 65, copyright 2018 Elsevier; (D) Schematic illustration of antigen presentation using antigen-encoding mRNA formulated with lipid nanoparticles. Reproduced with permission 66; (E) Schematic diagram of loading mRNA vaccines using self-assembled nanoparticles formulated with CP 2k. Adapted with permission from 67, copyright 2016 Elsevier; (F) Schematic representation of DP7-C modified liposomes enhancing immune response to neoantigen encoding mRNA complexes. Adapted with permission from 68, copyright 2020 Elsevier.

DCs are antigen-presenting cells (APCs) present in all tissues. DCs are primarily responsible for the uptake, internalization, and processing of antigens, and subsequently, they deliver processed antigen peptides to naïve T cells, serving as a bridge between innate and adaptive immunity 80. The presence of various specific receptors on the surface of DCs provides natural targets for the delivery vehicles aimed at targeting DCs 81. Here, we will discuss some different methods for DC-targeted delivery systems and related mRNA delivery systems (Figure 4A).

#### C-type lectin receptor (CLR) family

Many receptors used in targeted research belong to the CLR family. CLRs are a family of lectins that recognize carbohydrates in a calcium-dependent manner, and their carbohydrate recognition domains (CRDs) share primary structural homology 82. The N-terminus of the I-type CLR group is located extracellularly and mainly includes DEC205 (LY75/ CD205) and mannose receptor (MR/CD206/Clec13D). Most CLRs are of the II-type, with their amino terminus located intracellularly, which primarily includes Clec9A (CD370/DNGR-1) and DC-SIGN (Clec4L/CD209) 83.

MR is expressed on macrophages, endothelial cells, smooth muscle cells of the trachea, mature and immature moDC, and human peripheral blood CD1c^+^ DC 84. Kramer and colleagues prepared amphiphilic block copolymer micelles containing antigenic peptides and adjuvants through the hydrolysis of polymaleic anhydride (HPMA) and lauryl methacrylate to induce desired antigen-specific T-cell responses 85. Among them, mannose and trimannose are introduced into the hydrophilic corona as units targeting MR and DC-SIGN. Similarly, Zhu and colleagues developed mannose-functionalized lipid-hybrid polymersomes (MAN-IMO-PS) for co-delivery of ovalbumin antigen within the core, the hydrophobic membrane-embedded TLR7/8 agonist imiquimod, and the TLR4 agonist monophosphoryl lipid A on the lipid layer to induce synergistic anti-tumor immune responses 86.

Based on mannose, there have been a series of studies on targeted delivery of mRNA. Hybrid lipid-shell-polymer core mRNA nanoparticles (LPRs) may be valuable alternatives to lipid-based mRNA nanocomplexes (LRs), as they combine improved stability and reduced cytotoxicity 87. Therefore, researchers have developed the tri-mannosylated LPR nanoplatform to efficiently target the delivery of Fluc mRNA to DC cells (Figure 4B) 64. Subsequently, the authors validated the ability of this nano system to stimulate CD8 T-cell immune responses by delivering ovalbumin (OVA) and human papillomavirus 16 (HPV16) mRNA, achieving high anti-tumor efficacy. Moreover, the delivery of mRNA modified with N1-methylpseudouridine could reduce adverse inflammatory reactions. This study establishes that the LPR platform possesses excellent immunogenicity and improved inflammatory response modulation.

Human DC-specific intercellular adhesion molecule 3 grabbing non-integrin (DC-SIGN) is primarily expressed on the surface of immature dendritic cells (iDCs), with lower levels of expression on mature dendritic cells (mDCs) and macrophages 88. DC-SIGN has a CRD to bind ligands with high mannose and fucose structures, including Lewis(Le)-type antigens and host glycoproteins 89. DC-SIGN recognizes carbohydrate structures to facilitate antigen uptake, processing, and presentation through MHC II molecules, thereby enhancing T-cell responses 90. For example, research achieved specific DC-SIGN targeting by using a multivalent liposomal formulation containing the glycan Le^X^ to deliver adjuvants and tumor antigens to induce immune responses 91.

Based on DC-SIGN, Moignic group has demonstrated the capability of targeting with a lipid-polymer-RNA lipopolyplex functionalized with a tri-antenna of α-d-mannopyranoside (triMN-LPR), possessing binding affinity to DC-SIGN and CD207 (Langerin) on DCs (Figure 4C) 65. After intradermal injection of mRNA NPs encoding the papillomavirus E7 antigen to C57BL/6 mice, draining lymph nodes exhibited activated DCs, significant gene expression of CCR7 and CXCR4 at the injection site, and E7-specific T cell responses. In the E7-expressing TC1 tumor model, triMN-LPR NP resulted in significant long-term survival compared to control PBS injections.

One of the most well-known methods for targeting DCs is to use specific antibodies against DEC-205, which is expressed in both mouse and human DCs. DEC-205 has ten carbohydrate recognition domains with a molecular weight of 205 kDa 92. It is a phagocytic receptor that mediates antigen uptake and is highly expressed in DCs and thymic epithelial cells 92. To improve the selective distribution of nanoparticles, antibodies or other targeting moieties can be incorporated into the nanoparticles. For example, Katakowski *et al.* conjugated the reduced anti-DEC205 single-chain variable antibody fragments (scFv) to the maleimide groups of DSPE-PEG-MAL in LNPs by simple mixing 93. They demonstrated that the anti-DEC205 scFv-modified LNPs preferentially target DEC205^+^ DCs.

Clec9A is highly expressed in type 1 conventional dendritic cells (cDC1) in both humans and mice 94. Clec9A-targeted antigen delivery also promotes MHC-II antigen presentation to CD4^+^ T cells 94. Zeng *et al.* prepared functionalized nanoemulsions that encapsulated tumor antigens to target Clec9A (Clec9A-TNE), which can stimulate therapeutically effective tumor-specific immunity 95.

#### CD11c

In addition, some targeting approaches for DCs have been used for delivering drug/biological/antigen. Although they are not yet used for mRNA delivery, this could serve as inspiration for mRNA delivery. CD11c (CD18) is a type of leukocyte integrin receptor, which is primarily expressed on the surface of DCs 96. Antigens can be internalized through these receptors, facilitating antigen capture and processing by DCs 96. One study suggests that Fab fragments targeting CD11c conjugated with model antigen OVA can induce a significant T-cell response compared to other targets binding DCs, including CD205, TLR2 or FccRII/III 97. Another study indicates that encapsulating OVA antigen in PLGA-NPs modified with targeted antibodies, such as αCD40, αCD11c, and αDEC-205, can effectively enhance the internalization of NPs by DCs and IL-12 release 98. CD11c and DEC-205 receptors are considered to play a crucial role in the process of antigen capture and presentation and are almost exclusively expressed in DCs 99. Researchers anchored ScFv targeting DC markers, such as CD11c and DEC-205, to tumor-derived plasma membrane vesicles (PMVs) or lipid vesicles containing antigens, which can efficiently target the receptors on the surface of DCs 100.

#### Scavenger receptor (SRs)

SRs can recognize modified low-density lipoproteins (LDL), either through oxidized LDL or acetylated LDL. DC-asialoglycoprotein receptor (DC-ASGPR) is a lectin-like SR. Li and colleagues used DC-ASGPR to deliver self-antigens (PSA) and exogenous antigens (hemagglutinin 1, HA1) to DCs, leading to the generation of antigen-specific CD4^+^ T cell responses that produce IL-10 101. Scavenger Receptor Class B Type 1 (SR-B1) is one of the SRs that facilitates the uptake of cholesterol esters from circulating lipoproteins. Yuan and colleagues leveraged the high expression of scavenger receptor SR-B1 on mDCs and used nanoparticles (α-Ap-FNP) with SR-B1-targeting capabilities to directly transport tumor antigen peptides to DCs in lymph nodes, which resulted in significant inhibition of tumor growth 102.

#### Fc receptors (FcRs)

FcRs bind to the constant domains of antibodies, acting as connectors between humoral immune responses and cellular immune responses. FcRs are present in various immune cells, such as monocytes, macrophages, DCs, and neutrophils 103. Kawamura and colleagues used FcγR-targeted liposomes to deliver OVA, which was 2-5 times better than non-targeted liposomes 104. Similarly, another study showed that gold nanoparticles and liposomes targeted at DCs via FcγR (the receptor for the IgG Fc segment) were effective antigen delivery carriers, which can induce a stronger immune response compared to non-targeted nanoparticles or naked antigens 105.

#### Toll-like receptors (TLRs)

The immune system needs to distinguish between self-structures and foreign substances to detect pathogens and function. PRRs can distinguish self from non-self and recognize specific microbial-associated molecular patterns (MAMPs), which activate immune signals, acting as mediators between the innate and adaptive immune systems 106. TLRs are a type of PRR and are evolutionarily conserved proteins and are expressed on various APCs, such as DCs, B cells, and macrophages 107. Therefore, antigen-delivery vehicles modified with TLR ligands can generate excellent immune responses by targeting APCs and TLR-mediated APC stimulation. Li and colleagues designed cancer nanovaccines (BTs) prepared by fusing bacterial OMVs and tumor cell membranes (TCM) 108. Pathogenic adjuvants from bacteria can promote DC targeting, DC maturation, and antigen presentation.

#### Other DCs-targeting strategies based the physiochemical characteristics of NPs

With the advancement of nanotechnology, there have been extensive researches on optimizing the formulations of NPs to achieve the goal of DCs-targeted mRNA delivery 9. For example, Zhang *et al.* discovered LNPs with a 12-carbon tail, referred to as C1, which effectively delivers antigen-coding mRNA to DCs (Figure 4D) 66. Additionally, C1 can stimulate the expression of inflammatory cytokines in DCs by activating the TLR4 signaling pathway. This nano-vaccine exhibited promising anti-tumor efficacy *in vivo* therapies. Therefore, this minimalistic mRNA nano-vaccine offers a multifunctional platform for the development of personalized vaccines. Li *et al.* have developed a cationic cyclodextrin-polyethylenimine 2k conjugate (CP 2k) polymer-based intranasal mRNA vaccine delivery system for the treatment of HIV-1(Figure 4E) 67. The first cationic polymer capable of serving as a safe and effective intranasal mRNA vaccine carrier to overcome the nasal epithelial barrier has been provided. The authors observed significantly higher luciferase expression when transfecting CP 2k/mRNA (encoding luciferase mRNA) complexes into DC2.4 murine DCs, with an N/P ratio of 16, compared to using polyethylenimine (PEI) nanoparticles with a molecular weight of 25 kDa. Zhang *et al.* used a cholesterol-modified cationic peptide DP7 (VQWRIRVAVIRK)(DP7-C), with transmembrane structure and immunostimulatory properties to modify DOTAP liposomes, creating a universal mRNA delivery system (Figure 4F) 68. DOTAP liposomes modified with DP7-C (DOTAP/DP7-C) acted as carriers for mRNA and efficiently delivered mRNA to different types of DCs* in vitro*. As an immunostimulant, DOTAP/DP7-C liposomes were more effective than DOTAP liposomes in stimulating the maturation of DCs, the production of CD103^+^ DCs (which aids in antigen presentation), and the secretion of proinflammatory cytokines both *in vitro* and* in vivo*.

### mRNA NPs targeting T cells

**Figure 5:**
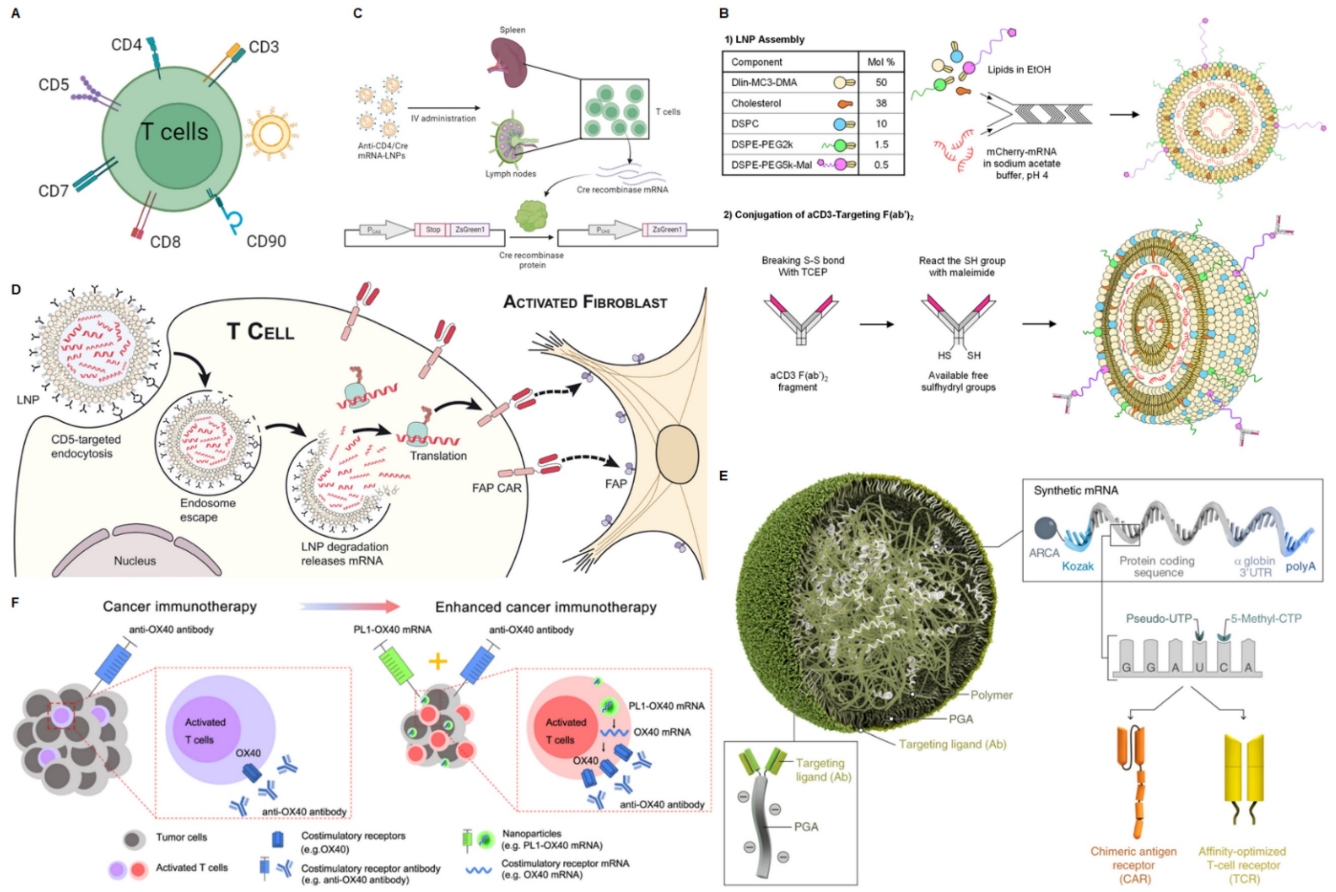
mRNA nanoparticles targeting T cells. (A) Schematic illustration of various receptors expressed on T cells that could be employed to design T cells-targeted NP-mediated delivery systems; (B) Schematic diagram of the preparation of mCherry mRNA for targeted delivery to T cells using αCD3 F(ab')_2_. Adapted with permission from 69, copyright 2022 Elsevier; (C) Schematic diagram of targeted delivery of mRNA to CD4^+^ T cells. Adapted with permission from 70; (D) Schematic representation of delivery of FAP CAR mRNA using CD5-targeted LNPs. Adapted with permission from 71, copyright 2022 American Association for the Advancement of Science; (E) Schematic representation of delivery disease-specific CAR or TCR transiently expressed nucleic acid (IVT mRNA) using CD8-targeted nanoparticles. Adapted with permission from 72, copyright 2020 Springer Nature; (F) Schematic illustration of enhanced tumor immunotherapy via phospholipid nanoparticles (PL1) delivery of OX40 mRNA combined with anti-OX40 antibodies. Adapted with permission from 73, copyright 2021 Springer Nature.

T cells are an integral component of the adaptive immune system, playing critical roles in defending against pathogen invasion, mediating anti-tumor immunity, establishing immunological memory, assisting B cells with antibody production, and regulating the activities of other immune cells. The activation and differentiation of T cells are precisely controlled by T cell receptor (TCR) signaling, costimulatory signals, and cytokines 109. By modulating the stimulatory signals, T cells can be “triggered” to produce perforin and granzymes to lyse tumor cells. The secretion of cytokines may induce tumor cell apoptosis through Fas-FasL interaction. Recent studies also reveal a new anti-tumor mechanism of T cells by promoting tumor ferroptosis 110. Activating endogenous T cells of cancer patients to suppress malignancy has been a central topic in tumor immunology research, but intracellular targeting of T cells remains a major challenge. Next, we will briefly discuss the targeting strategies for T-cell delivery (Figure 5A).

### CD3

The use of CD3-specific antibodies to target T cells provides an exciting approach to achieving T cell-specific delivery. Although anti-CD3 mAbs have been shown to deplete T cells while promoting *in vivo* anergy and extensive cytokine release, F(ab')_2_ fragments of anti-CD3ε can partially avoid this effect due to the lack of the Fc antibody portion 111. In addition, antibody engineering can optimize potency and affinity 112.

Based on the advantages of antibody engineering, CD3 bispecific antibodies have been extensively explored clinically and are a major research focus. Cheng *et al.* modified the surface of endogenous exosomes with two different scFVs, generating SMART-Exos that simultaneously target T cell surface CD3 and cancer cell-associated epidermal growth factor receptor (EGFR), which redirected and activated cytotoxic T cells to kill the cancer cells 113. Duwa and co-workers designed bis-R848-PLGA-NP containing dual-specific nanoparticles conjugated with two antibodies to sequentially target CD3 and the tumor-specific protein PD-L1 to enhance T cell cytotoxicity 114. Apart from that, there are also studies involving artificial APCs. Researchers utilize DC membranes with azide-functionalized loaded imiquimod and modify anti-CD3ε antibodies through click chemistry for stimulating T cells, which can potentiate cancer immunotherapy 115. Based on the CD3 target, there have also been many studies on targeted delivery of mRNA. For example, researchers evaluated the use of anti-CD3-targeted LNP for direct* in situ* transfection of T cells, rather than *ex vivo* administration of CD3 antibody or engineered therapeutic cells (Figure 5B) 69. Here, LNP packaged mCherry mRNA or Fluc mRNA in lipid-based nanoparticles and targeted T cells using αCD3 F(ab')_2_. *In vitro*, aCD3-LNP transfection infected and activated ~97% of Jurkat cells.* In vivo*, aCD3-LNP transfected 2-7% of circulating T cells and 2-4% of spleen T cells, causing transient activation, exhaustion, migration, cytokine release, and phenotypic changes. Huang *et al.* designed a novel liver-targeted ionizable lipid nanoparticle delivery system for mRNA encoding the B7H3×CD3 BiTE to exert potent anti-tumor activity against Acute Myelogenous Leukemia (AML) and melanoma 116.

#### CD4

CD4 is a transmembrane glycoprotein expressed on the surface of T helper cells. It serves as a co-receptor that assists the TCR in activating the T cell when bound to the β2 domain of major histocompatibility complex II (MHC II) on antigen-presenting cells. The presence of CD4 also contributes to T-cell signaling and trafficking 109. Therefore, CD4 is an appealing target for modulating T-cell-mediated immunity. Ramishetti *et al.* achieved specific delivery of siRNA to mouse CD4 T cells by modifying lipid nanoparticles with a monoclonal antibody against the CD4 receptor on the T cell surface, silencing CD45 by targeting T cells to deliver siCD45 117. McHugh *et al.* used anti-CD4 antibody-conjugated biodegradable nanoparticles loaded with TGF-β and IL-2 to induce the expansion of CD4^+^ Treg cells *in vitro* to directly improve clinical therapies for inflammatory and cell-mediated diseases 118.

Based on the CD4 target, Tombácz *et al.* achieved efficient, specific* in vitro* and *in vivo* mRNA delivery by using CD4 antibody-bound LNP to specifically target CD4^+^ cells and interfere with mRNA (Figure 5C) 70. After systemic administration in mice, radiolabeled mRNA-LNP accumulated in the spleen, providing a promising tool for *in vivo* T cell manipulation.

#### CD5

CD5 is a 67 kDa type I transmembrane glycoprotein belonging to the conserved scavenger receptor cysteine-rich (SRCR) family of receptors. It is also a pan-T cell marker, regularly expressed on normal T cells and about 85% of T cell malignancies, as well as some B cell malignancies 119. Researchers connected effector T cells expressing CD5 with B lymphoma target cells expressing CD19 using the bispecific antibody HD37xT5.16 (CD19xCD5), enabling activated effector T cells to interact with target cells and induce cell apoptosis 120.

Similarly, studies are achieving targeted delivery of mRNA to T cells through the CD5 target. Rurik and co-workers designed a CAR mRNA encoding for fibroblast activation protein (FAP, a marker of activated fibroblasts), and packaged it into CD5-targeted LNPs, termed “targeted antibody/LNP-mRNA cargo” or CD5/LNP-FAPCAR) (Figure 5D) 71. CD5 is naturally expressed by T cells and a small subset of B cells and is not required for T cell effector function 121. Treatment with the targeted modified mRNA LNPs reduced fibrosis and restored cardiac function after injury 71.

#### CD8

CD8 is a transmembrane glycoprotein that serves as a co-receptor for the TCR. It is expressed on the surface of cytotoxic T cells. The CD8 co-receptor binds to MHC class I molecules on antigen-presenting cells to recognize and kill cells displaying antigenic peptides bound to MHC class I. After TCR activation, CD8^+^ cytotoxic T cells release perforin, granzymes, and cytokines like IFN-γ to induce apoptosis of the target cell. Therefore, CD8 plays a crucial role in the cytotoxic function and anti-tumor immunity mediated by CD8^+^ T cells. Schmid and co-workers targeted compound delivery to specific leukocyte subpopulations to enhance the therapeutic index using anti-CD8a F(ab')_2_ fragments against CD8 T cells 122. These modified nanoparticles could bind to CD8^+^ T cells and deliver drugs to play a better anti-tumor role. In addition, Parayath *et al.* reported injectable nanoparticles for delivery of *in vitro* transcribed (IVT) CAR or TCR mRNA to reprogram circulating T cells to recognize disease-associated antigens (Figure 5E). Surface-anchored targeting ligands selectively bind nanoparticles to T cells and initiate rapid receptor-induced endocytosis for their internalization, which is achieved by conjugating anti-CD8 antibodies with polyglutamic acid (PGA) 72. The resulting mRNA nanocarriers could be lyophilized for long-term storage.

#### Other targeting strategies

Besides the markers expressed on the surface of T cells described above, some other molecules specifically expressed on T cells including CD7^123^, CD90 (Thy1.1) 124. For example, Lee* et al.* used chitosan nanoparticles modified with scFvCD7 to deliver siCD4 to T cells to enhance the ability to bind to T cells and higher silencing efficiency of CD4 compared to unmodified chitosan nanoparticles 125. Zheng *et al.* used PEGylated liposomes targeted respectively to the unique cell surface antigen CD90 on transferred T cells, which provided highly specific targeting with liposome binding to over 90% of cells after a single injection 126. Similarly, researchers prepared PEGylated immunoliposomes delivering the TGF-β inhibitor SB525334 (TGF-βI) and compared targeting using the internalizing receptor CD90 versus the non-internalizing receptor CD45 to achieve T cell-targeted delivery 127. These potential targets used for targeted delivery of other therapeutic agents can serve as references for future targeted delivery of mRNA to T cells.

#### Other T cell-targeting strategies based the physiochemical characteristics of NPs

Besides the strategies based on the ligand-receptor interaction described above, there are also some T cell-targeting delivery strategies based on inherent physiochemical characteristics of NPs. Researchers have shown that maleimide-functionalized NPs covalently coupled to free thiol groups on T cell membrane proteins can effectively deliver compounds to T cells 128. For example, Dong *et al.* designed a biomimetic phospholipid nanoparticle, PL1, which could deliver the costimulatory receptor OX40 to T cells and synergize with an agonistic anti-OX40 antibody to mediate effective antitumor treatment (Figure 5F) 73. Based on the chemical structure of natural cell membrane components, phospholipids, and glycolipids, the authors designed and synthesized a library of biomimetic materials comprising biomimetic heads (phosphate or sugar heads), ionizable amino cores, and multiple hydrophobic tails. The screened PL1 nanoparticles not only efficiently delivered co-stimulatory receptor mRNA to T cells *in vitro* but also effectively delivered it to T cells within tumors* in vivo*, providing valuable delivery materials for modulating T cell function.

### mRNA NPs targeting NK cells

**Figure 6:**
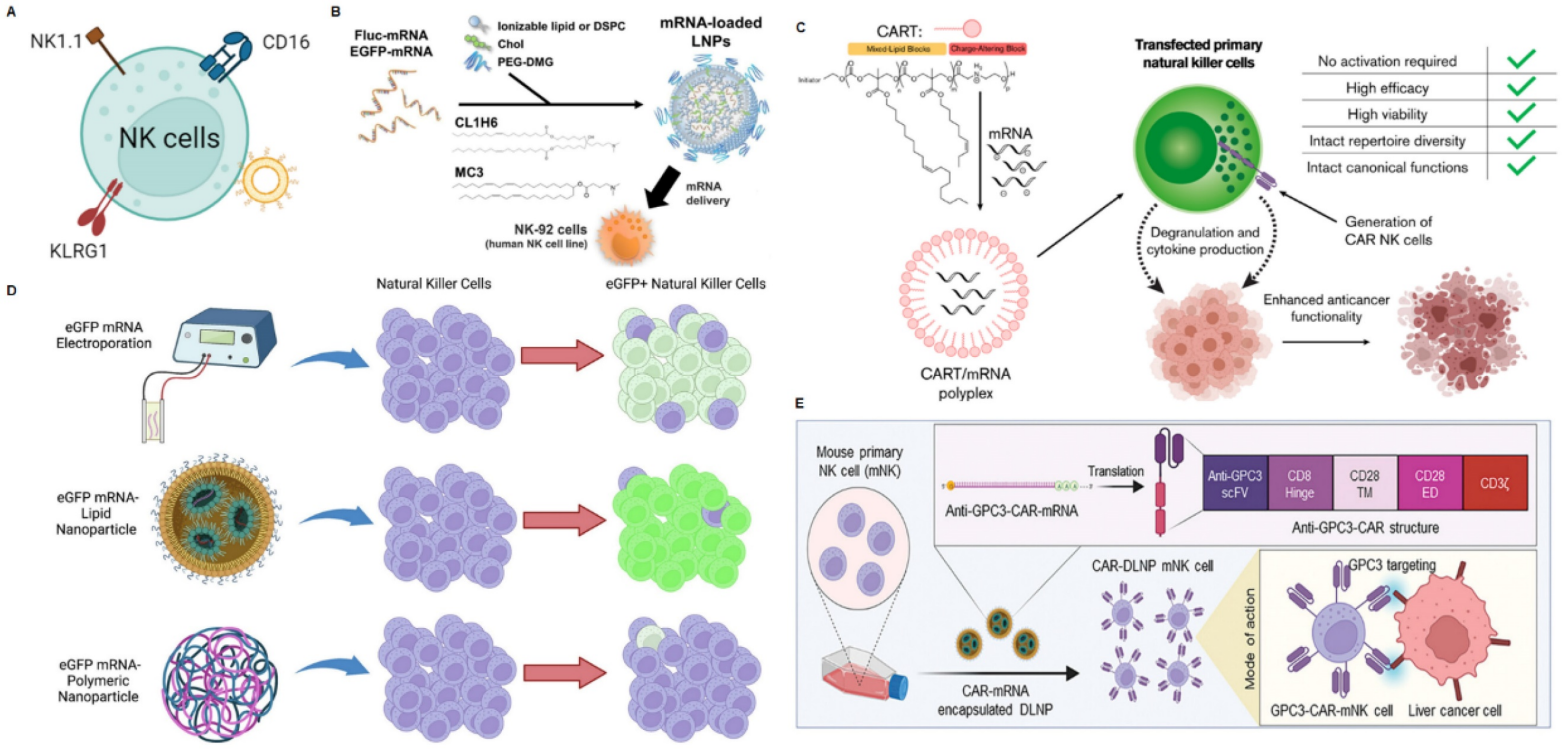
mRNA nanoparticles targeting NK cells. (A) Schematic illustration of various receptors expressed on NK cells that could be employed to design NK cells-targeted NP-mediated delivery systems; (B) Structural schematic of CL1H6-LNP for mRNA delivery. Adapted with permission from 137, copyright 2023 Elsevier; (C) Mechanism schematic and advantages of CART for targeted delivery of mRNA to NK cells. Adapted with permission from 138, copyright 2020 Elsevier; (D) Optimized LNP-based mRNA targeting NK cells showed higher transfection efficiency and higher overall eGFP expression than electroporation or polymeric nanoparticles. Adapted with permission from 139, copyright 2023 Elsevier; (E) Schematic representation of the process of generating anti-GPC3-CAR NK cells in mouse primary NK cells (mNK cells) through DLNPs-mediated transfection of mRNAs 74, copyright 2024 John Wiley and Sons.

NK cells, as an important component of the innate immune system, play a vital role in eliminating senescent cells and pathogenic microbes. With tumor cells downregulating MHC expression to evade adaptive immunity, they become more susceptible to NK cell cytotoxicity 129. Mechanistically, NK cells play a critical role in the first line of defense against cancer, mediating antitumor effects via two pathways: direct cytotoxicity by releasing perforin and granzymes or antibody-dependent cell-mediated cytotoxicity (ADCC) through death receptor ligands 130. NK cells also participate in tumor cell clearance by secreting cytokines or mobilizing DCs, macrophages, T cells, and other immune cells, which are emerging as promising candidates to be attractive targets for cancer immunotherapy 131. Here, we will discuss some different methods for NK-targeted delivery systems and related mRNA delivery systems (Figure 6A).

Cluster of differentiation 16 (CD16/FcγRIIIA), NK1.1, killer cell lectin-like receptor G1 (KLRG1) are specifically expressed in NK cells and have been used as targets for targeted delivery, providing reference significance for targeted delivery of mRNA. Based on CD16, researchers developed a bispecific Au nanoparticle that was dual-conjugated with IgG anti-HIVgp120 and IgG anti-human CD16 antibodies to enhance intercellular contact between HIV-expressing cells and NK cells 132. To further enhance NK cell-mediated antitumor activity, trifunctional NK cell engagers (NKCEs) have recently been designed and produced. A study reported a trispecific NKCE platform, including α-CD16, α-4-1BB, α-EGFR and epirubicin (EPI), which facilitated the recruitment and activation of NK cells, which can ultimately promote NK cell recruitment and activation to eradicate these cancer cells 133. Based on NK1.1, Chandrasekaran *et al.* designed functionalized liposomes for NK cells, which were decorated with anti-mouse NK1.1 antibody and tumor necrosis factor-α related apoptosis-inducing ligand (TRAIL) that initiated cell apoptosis through interacting with death receptors on cancer cells 134. Based on KLRG1, Jiang *et al.* developed an NK cell-targeting immunomodulating nano-adaptor (imNA) to promote NK cells engagement of tumor cells for better exertion of tumor cytotoxicity, including αFc-NP with anti-KLRG1 antibody and anti-PDL1 antibody 135. Compared to free antibodies, this strategy significantly reduced lung metastatic melanoma formation.

Current researches on targeting NK cells to deliver mRNA are mainly based on the physical and chemical characteristics of nanoparticles. For example, Nakamura and co-workers developed an LNP composed of CL1H6 (CL1H6-LNP) for effective siRNA delivery to NK-92 cells, achieving low cytotoxicity and efficient gene silencing 136. Subsequently, they used CL1H6-LNP to deliver mRNA to NK cells (Figure 6B) 137. The study indicated that LNP exhibited significantly higher mRNA expression intensity, primarily attributed to its high affinity with NK-92 cells and rapid, robust fusion with endosomal membranes. Thus, CL1H6-LNP serves as a non-viral vector that can regulate the function of NK-92 cells by delivering mRNA and thereby promote tumor immunotherapy. Wilk and co-workers reported that a cost-effective, easily synthesizable non-viral charge-altering releasable transporter (CART) can effectively transfect mRNA into primary human NK cells, independent of NK cell activation (Figure 6C) 138. Compared to electroporation, CART is more efficient in transfecting NK cells, better preserves cell viability, and minimizes reshaping of NK cell phenotype and function. More importantly, the authors produced anti-CD19 human CAR NK cells *in vitro* by CART-mediated transfection of mRNA encoding anti-human CD19-41BB-CD3ζ CAR (hCAR). These CAR NK cells exhibited potent cytotoxicity and enhanced activation of CD19^+^ target cells compared to their untransfected counterparts. This predicts that CAR NK prepared by CART-mediated mRNA transfection greatly facilitates tumor immunotherapy. Douka prepared polymer and LNPs for the delivery of enhanced green fluorescent protein (eGFP)-mRNA into NK cells, including triblock *co*-polymer pHDePA, the homopolymer pHPMA-DEAE, and PEGylated forms of pDMAEMA (Figure 6D) 139. By optimizing the lipid components and mRNA encapsulation methods, a promising lipid-complex-based mRNA formulation for NK cell transfection was identified, successfully delivering eGFP-mRNA to KHYG-1 cells. This NP-based mRNA delivery is a promising strategy for further development of novel NK cell therapies. For example, NK cells were engineered to overexpress CAR mRNAs encoding activating receptors (CD16 or CXCR4) to enhance their tumor targeting and cytotoxicity, thereby facilitating tumor immunotherapy 140. Recently, it was reported that a DOTAP-functionalized lipid nanoparticle could deliver CAR mRNA to NK cells via lectin-mediated endocytosis and enhanced the ability of killing tumor cells via the extracellular signal-regulated kinase/Mitogen-Activated Protein Kinase pathway modulation and mitochondrial dynamics changes 74. More importantly, the study achieved therapeutic effects of orthotopic HCC tumor model by injecting mouse-derived engineered anti-glypican 3 (GPC3)-CAR-NK cells 74. The strategy for modifying CAR-NK enhance tumor immunotherapy.

### mRNA NPs targeting macrophages

**Figure 7:**
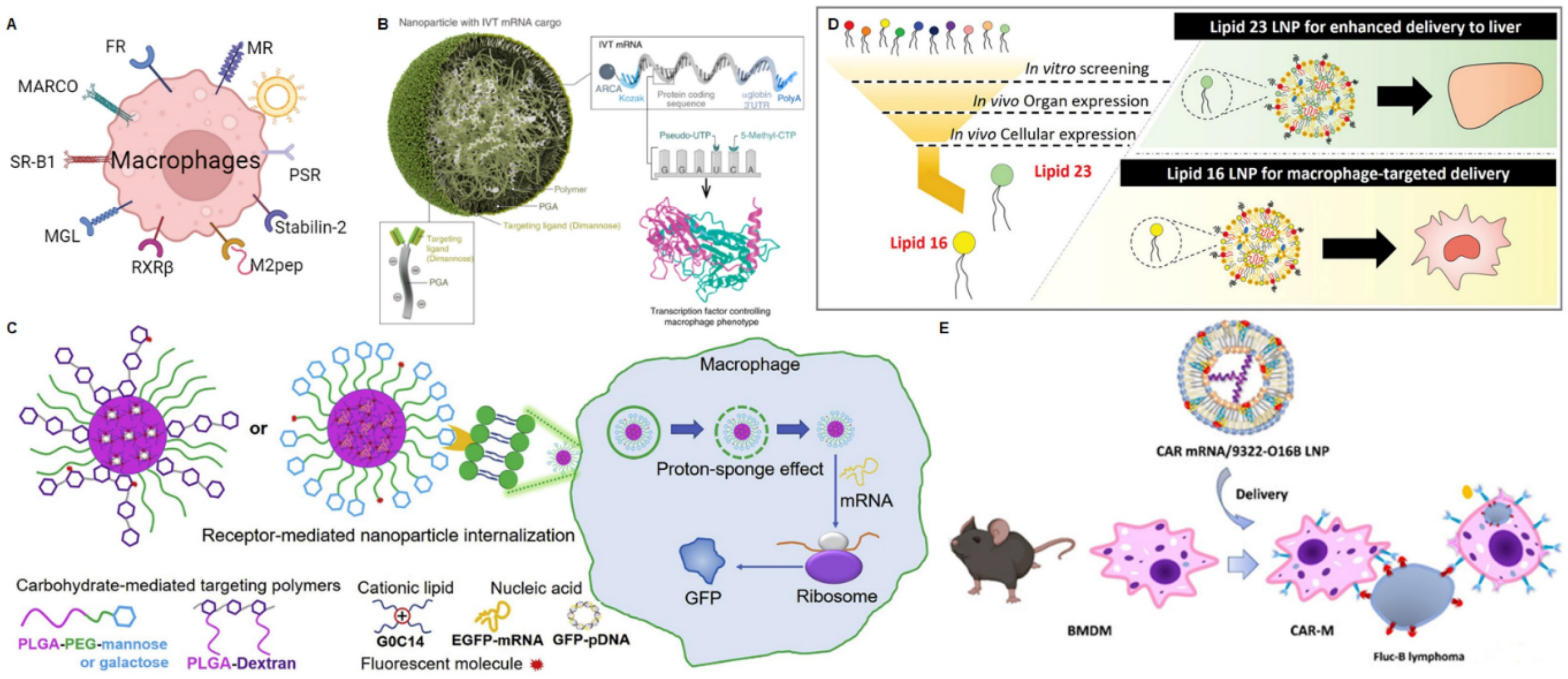
mRNA NPs targeting macrophages. (A) Schematic illustration of various receptors expressed on macrophages that could be employed to design macrophages-targeted NP-mediated delivery systems; (B) Schematic illustration of nanoparticles with mannose receptor targeting loaded with mRNA encoding reprogramming transcription factors. Adapted with permission from 75, copyright 2019 Springer Nature; (C) Schematic diagram of the structure and mechanism of action of a series of macrophage-targeting nanoparticles with different carbohydrate modifications; Adapted with permission from 150, copyright 2020 Elsevier; (D) Schematic of screening and structural of LNPs with macrophage targeting and liver targeting. Adapted with permission from 76, copyright 2023 John Wiley and Sons; (E) Schematic illustration of lipid nanoparticles encapsulating CAR mRNA for targeted delivery to macrophages. Adapted with permission from 169, copyright 2022 American Chemical Society.

Macrophages are a type of white blood cell that belong to the innate immune system and play various roles in maintaining tissue homeostasis 141. Macrophages have two main phenotypes and the balance between M1 and M2 macrophages in the tumor microenvironment is crucial. Tumors often exploit the immunomodulatory functions of macrophages to create an immune-suppressive environment that supports their growth and survival. Strategies aiming to repolarize tumor-associated macrophages into the M1 phenotype have been studied as a potential approach to enhance anti-tumor immune responses 142. As the non-specific delivery method could inevitably lead to adverse systemic effects, the strategy commonly used by researchers is to use cell surface markers to modify specific targeting ligands for the targeted delivery of therapeutic agents. Examples of TAM markers widely used for ligand-targeted delivery include mannose receptor (MR), folate receptor (FR-β), and scavenger receptor (MARCO, SR-B1) (Figure 7A) 143, 144.

#### Mannose receptor (MR)

MR is a type I transmembrane protein belonging to the C-type lectin family and is primarily located on the surface of macrophages and iDCs 145. The expression of MR is further upregulated when these monocytes extravasate from the circulation and are exposed to factors in the tumor microenvironment around blood vessels 146. Due to the high expression of CD206 in TAMs, strategies that optimize cargo absorption through these receptors can be highly effective. Li* et al.* synthesized mannose-modified porous hollow iron oxide nanoparticles (PHNP) for loading the PI3Kγ small molecule inhibitor (3-methyladenine, 3-MA) to target TAMs and activate the immune response 147. Chen *et al.* developed a series of PEG conjugate nanocarriers with varying numbers of mannose units and identified the optimal structural configuration for targeting macrophage-like J774.E cells 148. Ye *et al.* established a siRNA conjugate platform to reduce the expression of Marburg virus (MARV) for prolonged survival benefit 149. This nanoplatform contains a hexavalent mannose conjugate that can target macrophages and DCs, and GalNAc-siRNA conjugates that can achieve liver cell targeting through the asialoglycoprotein receptor (ASGPR).

Based on the discussed targeted techniques for macrophages, various mRNA delivery systems targeting macrophages have been developed. Zhang described a TAM-targeted nanoparticle that can deliver *ex vivo* transcribed mRNA encoding M1 program transcription factors to induce anti-tumor immunity (Figure 7B) 75. These particles are composed of polyglutamic acid (PGA) functionalized with Di-mannose coated mRNA-PbAE complexes, which target macrophages expressing the MR with an M2-like phenotype. Meanwhile, nanoparticles encoding mRNA for interferon regulatory factor 5 (IRF5) significantly reduced tumor progression in ovarian cancer, melanoma lung metastasis, or glioblastoma models.

Chen *et al.* prepared a series of nanoparticles targeting macrophages using cationic lipid compounds G0-C14 and different carbohydrate modifications on poly(lactide-co-glycolide) (PLGA) or poly(lactide-co-glycolide)-*b*-poly(ethylene glycol) (PLGA-PEG) through self-assembly (Figure 7C) 150. The carbohydrate modifications include mannose, lactose, maltose, and a mixture of mannose and lactose. EGFP messenger RNA (mRNA) was used as reporter genes to assess NP-mediated gene transfection in macrophages. Macrophage engulfment experiments showed that more carbohydrate-modified nanoparticles were internalized by Raw 264.7 cells compared to nanoparticles without carbohydrate modification. Compared to nanoparticles modified with other carbohydrate modifications, mannose-modified nanoparticles exhibit superior targeting capability to macrophages. This provides a potential technical platform for delivering biologics and therapeutic genes to macrophages in inflamed areas. Recently, Tang *et al.* also discovered a dual-targeting nano-delivery system that simultaneously targets pulmonary macrophages and tumor cells by DSPE-PEG-Mannose and HA for mRNA delivery 151. This finding provides guidance for the development of vaccines or drugs for pulmonary-related diseases.

#### Folate receptor (FR)

In addition to the examples mentioned above, researchers have also identified other potential targeting ligands for macrophage-targeted mRNA delivery. FR is a 38 kDa glycosylphosphatidylinositol (GPI)-anchored protein 152. The FR family comprises four members, including FRα (FOLR1), FRβ (FOLR2), FRγ (FOLR3), and FRδ (FOLR4). FRα and FRβ are anchored to the cell membrane via GPI, and they are overexpressed in tumor cells and TAMs 153. A study indicated that folate can be used to target TAMs in a murine ovarian cancer xenograft model via liposomes 154. Another study found that the overexpression of FRβ in TAMs is associated with poor prognosis in lung cancer. Therefore, the author utilized folate-modified liposomes (F-PLP) to deliver a plasmid containing BIM-S (a cell death mediator that interacts with BCL-2) to target lung cancer cells and FRβ-positive macrophages in the tumor microenvironment for the significant suppression of* in vivo* tumor growth 155.

#### Scavenger receptors (SR)

Macrophage surface scavenger receptors primarily include the following categories: Class A SR, mainly SR-A1 and macrophage receptor with collagenous structure (MARCO), which can recognize the lipopolysaccharides of Gram-positive bacteria; Class B SR, mainly CD36 and SR-B1, which can recognize oxidized LDL 156. These receptors are widely distributed on the surface of macrophages and play a crucial role in the biological functions of macrophages by the recognition of various ligands.

MARCO has been identified as a gene that is overexpressed in the tumor microenvironment (TME) and is associated with poor prognosis in human breast cancer 157. A study indicated that the anti-MARCO antibody has the potential to repolarize MARCO-positive TAMs 158. Another study found that negatively charged immune-modifying microparticles (IMPs) can be taken up by inflammatory monocytes via the MARCO on macrophages 159.

SR-B1 participates in the phagocytosis of apoptotic cells by recognizing oxidized phospholipids displayed on the cell membrane when cells undergo apoptosis 160. Wang and colleagues synthesized high-density lipoprotein-mimetic peptides-phospholipid scaffolds (HPPS) modified with apoA-1 mimetic peptide (R4F) that could target peripheral monocytes* via* the SR-B1 receptor 161. Kuninty and co-workers designed engineered nanoliposomes containing peroxidized phospholipids that could be recognized and internalized by the SR-B1 for the delivery of STAT6 inhibitor (AS1517499), zoledronic acid, or cell wall lipopeptides to inhibit the premetastatic microenvironment and tumor growth 162.

#### Other targeting ligands

Besides the targeted ligands described above, some other molecules specifically expressed on macrophages including macrophage galactose-type lectin (MGL) 163, retinoid X receptor β (RXRβ) 164, stabilin-2 165, and phosphatidylserine receptors (PSR) 156, have been employed as targeting ligands to design and develop macrophage-targeted NPs-based delivery systems. For example, based on the specific recognition between peptide sequence CRTLTVRKC (denoted S2P peptide) and stabilin-2 166, Tao *et al.* recently developed a S2P peptide-modified polymeric NPs for macrophage-targeted delivery of Camk2γ siRNA and the treatment of atherosclerosis 167. Although these macrophage-targeted delivery systems have not been designed for mRNA delivery, they definitely provide the potential strategies and guidelines for the future development of macrophage-targeted mRNA delivery techniques.

#### Other macrophage-targeting strategies based the physiochemical characteristics of NPs

In addition to the ligand-receptor interaction to achieve macrophage-targeted mRNA delivery, it has been found that the macrophage-targeted mRNA delivery could be also achieved by optimizing the physiochemical characteristics of NPs. For example, Naidu *et al.* synthesized a new library of ionizable lipids by modifying the hydrophobic tails and linker regions, incorporating other helper lipids, and using microfluidic mixing techniques to form stable LNPs (Figure 7D) 76. Subsequently, they conducted rigorous* in vitro* and *in vivo* screening experiments to identify lipids suitable for cell-type-specific targeting of macrophages (Lipid 16) and high-quality lipids for liver targeting (Lipid 23). This study demonstrated that their structural modules could drive cell-specificity, opening new pathways for the development of efficient mRNA therapies using amino-lipid-based LNPs. The FDA approval of two anti-CD19 chimeric antigen receptor (CAR) T cell therapies has spurred increased research interest in CAR therapies 168. Therefore, Ye *et al.* screened for optimal mRNA and lipid formulations to deliver mRNA coding for anti-CD19 CAR into primary mouse macrophages for the treatment of B-cell lymphoma (Figure 7E) 169. The authors utilized the RAW264.7 cell line as a macrophage model and identified an optimized LNP formulation (9322-O16B/Chol/DOPE, 16:10:1, w/w) for delivering anti-CD19-eGFP mRNA. This improved CAR mRNA delivery system holds potential clinical applications for cell therapy.

### mRNA NPs targeting B cells

B cells can regulate immune function by recognizing antigens, differentiating into plasma cells, producing antigen-specific antibodies, acting as APCs, and secretion of cytokines 170. B cell dysregulation is associated with autoimmune diseases such as systemic lupus erythematosus, rheumatoid arthritis, and cancer 171. Hence, the use of nanoparticles to modulate B cells is a promising strategy.

**Figure 8:**
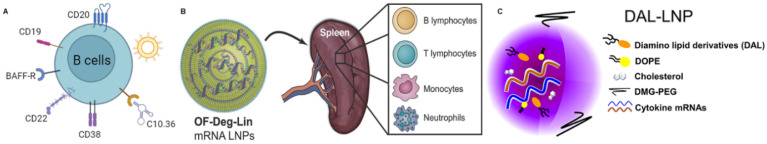
mRNA NPs targeting B cells. (A) Schematic illustration of various receptors expressed on B cells that could be employed to design B cell-targeted NP-mediated delivery systems; (B) Schematic diagram of OF-Deg-Lin mRNA LNP targeting various immune cells including B lymphocytes in the spleen; Adapted with permission from 77, copyright 2017 John Wiley and Sons; (C) Schematic diagram of DAL-LNP loaded with mRNA. Adapted with permission from 78, copyright 2022 Elsevier.

Currently, the targeting strategies for B cells primarily rely on receptors that are specific to B cell expression, including CD20 172, CD19 173, B-cell activating factor receptor (BAFF-R) 174, CD22 175, CD38 176, *etc*. (Figure 8A)*.* For instance, poly(lactic-co-glycolic acid) (PLGA) nanoparticles with a core coated with a poly-L-arginine layer and dual-targeting outer layers of CD20 and CD44 antibodies have been developed for delivering siBCL-2 to lymphoblastic leukemia and B lymphoma cells 177. Satake and co-workers discovered that the conjugation of superparamagnetic iron oxide nanoparticles (SPIO NPs) with anti-CD22 mAb enhances the delivery efficacy of siRNA therapy in acute lymphoblastic leukemia (ALL) 178. Puente and co-workers reported the first study using CD38 as a target for a drug delivery system to treat multiple myeloma (MM) 179. They modified polymer chitosan nanoparticles loaded with bortezomib (BTZ) with αCD38 mAb to enhance targeting efficacy and therapeutic outcomes.

Aptamers are a class of single-stranded oligonucleotides showing high specificity 180. In addition, aptamers are generally easier to obtain than antibodies with low expense and high stability. Researchers reported a modular nanostructure that delivers fluorescent RNA aptamers (50-80 kDa, 175-250 nt) to target cells by recognizing various human B cell cancer cell lines and transferrin receptor-expressing cells 181. The C10.36 aptamer is a compact G-quadruplex DNA that internalizes into B-cell cancer cell lines upon binding to an unknown cell surface molecule 182. Although these B cell-targeted delivery systems were not designed for mRNA delivery, they undoubtedly provide potential strategies and guidelines for the future development of B-cell-targeted mRNA delivery technology.

Based on the physical and chemical characteristics of nanoparticles, there are also some applications in the research of B-cell-targeted mRNA delivery. Fenton and co-workers designed a LNP delivery system that can encapsulate mRNA and transfect B lymphocytes in the spleen, inducing protein expression in B cells (Figure 8B) 77. While LNPs can be transiently observed in the liver and other organs, this LNP can induce the expression of more than 85% of the protein in the spleen. These results suggest that OF-Deg-Lin mRNA LNPs, as delivery vehicles, can significantly enhance protein expression in B lymphocytes in the spleen. It also demonstrates the significant advantages of nanomaterials in achieving organ and cell targeting. Liu *et al*. developed a novel mRNA delivery vehicle, DAL4-LNP, for delivering cytokines mRNA (Figure 8C) 78. By intratumorally injection of DAL4-LNP loaded with GFP-coding mRNA (DAL4-LNP-GFP) into B16F10 tumors, the authors observed that LNP can selectively deliver mRNA targeting to CD19 B cells. By flow cytometry analysis, approximately 98% of GFP-positive immune cells are B cells.

### mRNA NPs targeting neutrophils

**Figure 9:**
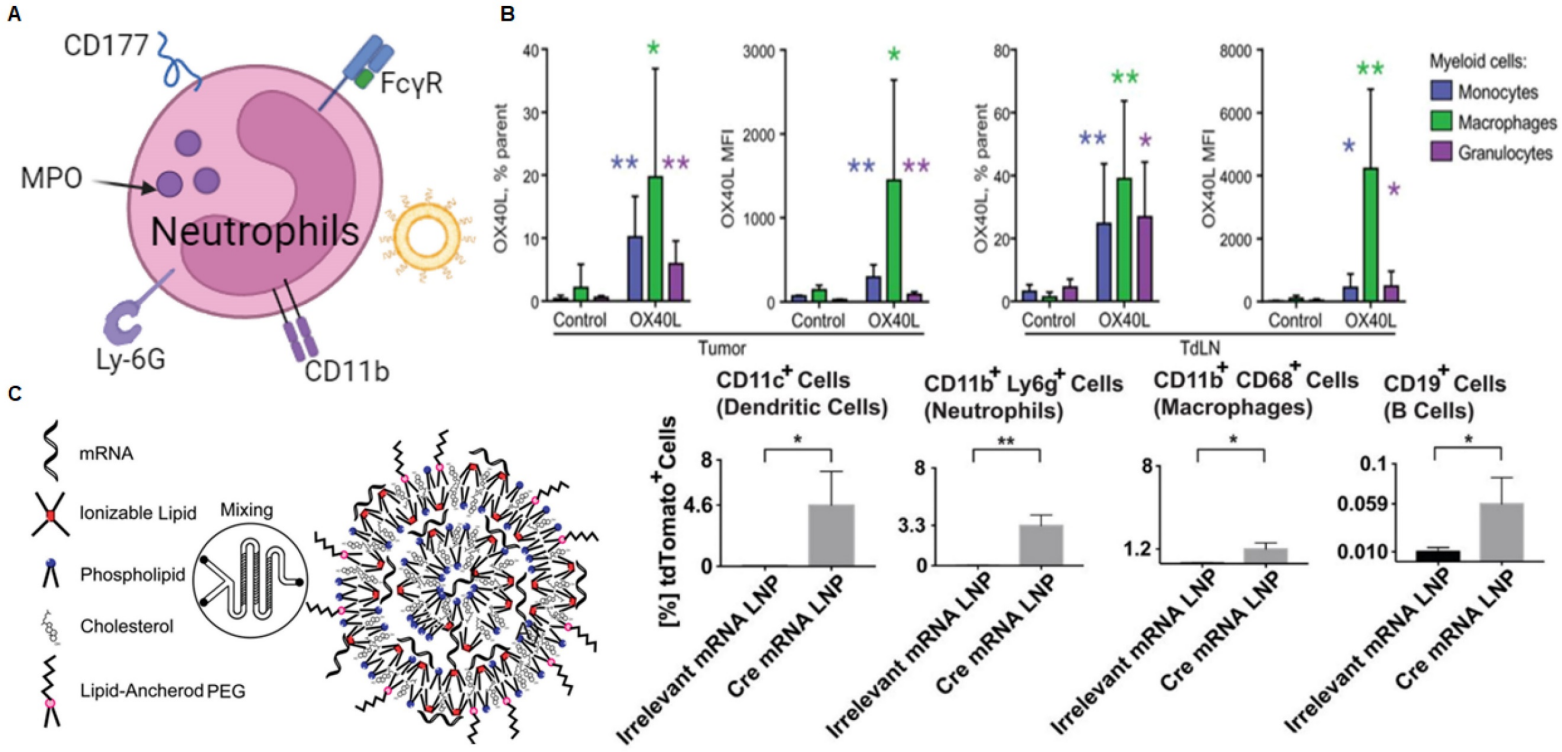
mRNA nanoparticles targeting neutrophils. (A) Schematic illustration of various receptors expressed on neutrophils that could be employed to design neutrophils-targeted NP-mediated delivery systems; (B) After intratumoral injection of 5 μg of mRNA encoding OX40L, OX40L mRNA can be delivered to a variety of bone marrow cells, including granulocytes. Adapted with permission from 31, copyright 2019 The American Association for the Advancement of Science; (C) Schematic diagram of the synthesis of lipid nanoparticles containing mRNA and the expression of Cre mRNA in various immune cells including neutrophils. Adapted with permission from 79, copyright 2017 American Chemical Society.

Granulocytes make up the largest proportion of white blood cells. They enter the bloodstream and survive for several hours before leaving and dying 183. Granulocytes include eosinophils, neutrophils, and basophils and are distinguished by granule staining. Neutrophils are a type of phagocyte found in the blood and are one of the first responders in the early stages of inflammation caused by bacterial infection and environmental changes 184. They can be rapidly recruited to sites of tissue damage and exert antibacterial and inflammatory functions through phagocytosis, degranulation, neutrophil extracellular traps (NETs), and antigen presentation 185. Therefore, targeting neutrophils could be a new therapeutic approach to treat inflammatory diseases and cancer (Figure 9A).

Some other molecules specifically expressed on neutrophils, including Fcγ receptor III (FcγRIII) 186, CD177 187, Myeloperoxidase (MPO) 188, Ly-6G 189, CD11b (integrin αM, called Mac-1) 190. For example, Wang *et al.* internalized resveratrol-loaded albumin particles into neutrophils adhering to inflamed endothelial surfaces through the FcγRIII receptor, which can be used to prevent lung injury 191. Researchers have utilized phage display technology to identify peptide sequences that specifically bind to CD177. They found that modifying liposomes with neutrophil-specific peptides enhances neutrophil-specific delivery and the ability to alter neutrophil function, thus potentially treating various diseases 192. Tang *et al.* self-assembled a ligand known as bis-5HT, which has two serotonin (5-hydroxytryptamine or 5-HT) terminal ends, with poly(propylene glycol)-poly(ethylene glycol)-carboxyl (PLGA-PEG-COOH) to create MPO and neutrophil targeting nanoparticles to improve tumor therapy^193^. Although these neutrophil-targeted delivery systems are used to deliver other therapeutic agents, they provide potential strategies and guidelines for the future development of neutrophil-targeted mRNA delivery technology.

While there are few reports on neutrophil-targeted mRNA delivery, various combinations of cytokine therapy and LNP-related mRNA vaccines have been confirmed to deliver mRNA to neutrophils. A study described a delivery system encapsulated in LNP for intratumoral delivery of IL-23/IL-36γ/OX40L trimeric mRNA, which can achieve complete remission (CR) of tumors (Figure 9B) 31. Specifically, after intertumoral injection of 5 μg of mRNA encoding OX40L, the authors observed the expression of OX40L in three of the most abundant bone marrow cells, including macrophages, monocytes, and granulocytes. This suggests that the strategy can effectively deliver mRNA to granulocytes to exert its effects. Similarly, researchers developed LNPs for mRNA vaccine delivery to induce CD8 T-cell cytotoxicity (Figure 9C) 79. Specifically, the authors found that the screened B-11 LNPs can be taken up by various immune cells, including DCs, macrophages, neutrophils, and B cells. Flow cytometry analysis revealed that 3.3% of neutrophils in the inguinal lymph nodes expressed the mRNA.

## Clinical status of mRNA-based therapeutics

To date, there have been more than 1,000 clinical trials of mRNA-based cancer therapy. LNPs account for a large portion of these, most of which are in clinical phases I and II. We summarize the results of the current clinical trials of mRNA therapy, which suggest that mRNA therapy will be a promising strategy, and that further research and development will advance antitumor therapy (Table 5).

## Conclusion, prospects, and challenges

Immunotherapy has revolutionized cancer treatment, including therapies like immune checkpoint inhibitors (ICI) and adoptive cell therapies. However, due to tumor heterogeneity, patient's benefit rates need to be improved. Furthermore, short half-lives of immunotherapeutic agents and adverse autoimmune reactions pose significant challenges in cancer immunotherapy. Recent studies have highlighted the therapeutic potential of mRNA therapy in various applications. However, challenges such as mRNA instability and immunogenicity must be addressed to enhance mRNA effectiveness. Therefore, the selection of suitable mRNA delivery vehicles is crucial. Furthermore, for the treatment to be effective, mRNA molecules must reach target cells and produce sufficient target proteins. Overcoming various biological and pharmacological obstacles in clinical applications is essential. Targeted delivery plays a crucial role, and guiding the delivery of mRNA macromolecules to immune cells using nanoparticles is significant, including T cells, DCs, NK cells, macrophages, B cells, and neutrophils. In this review, we systematically summarize existing mRNA targeting delivery nanoparticles for immune cells and potential methods for mRNA delivery targeting. This aims to enable the production of long-lasting therapeutic drugs while minimizing off-target toxicity to the greatest extent.

mRNA nanotechnology-mediated immunotherapy has been widely applied in preclinical and clinical research for cancer treatment. Recent achievements highlight the potential of mRNA as a breakthrough treatment for various disease. Despite the promising prospects and remarkable success of mRNA technology in cancer immunotherapy, there are challenges in advancing mRNA as a therapeutic immune drug in clinical practice. Firstly, it is essential to deepen our understanding of the relationship between modified mRNA constructs and their stability, translation, and immune regulation. High-throughput methods are attractive strategies that can establish correlations between mRNA performance and its numerous structural variants accurately and rapidly. Additionally, tumors have complex microenvironments and heterogeneity, and intercellular interactions affect tumor progression. Emerging techniques such as single cell sequencing and spatial transcriptomics help us understand the interaction mechanisms between mRNA and the patient's TME, providing a basis and support for developing the next generation of mRNA nanoparticles. Furthermore, optimizing mRNA delivery platforms is necessary. mRNA delivery platforms enhance the stability of exposed mRNA. Designing mRNA delivery platforms with high loading capacity and enhanced mRNA translation efficiency is crucial for improving safety and efficacy. In the context of targeting immune cells in tumor sites, nanoparticles encapsulating mRNA encoding immune-stimulating proteins can be passively delivered to tumors through the enhanced permeability and retention (EPR) effect. However, their effectiveness is often limited by multiple physical barriers. Therefore, designing nanoparticles that actively target mRNA molecules to specific cells with high efficiency is paramount. It is worth noting that the physicochemical properties of these biomaterials may affect the activation of immune pathways, or these interactions might alter the function of tissues or cells involved in immune regulation, which remains an unresolved issue. It's worth noting that introducing targeting modules into NPs typically involves multiple steps of synthesis, purification, and characterization, significantly increasing the complexity, cost, and regulatory challenges of production. Furthermore, the targeting capability of functionalized nanoparticles in a biological environment may be diminished or lost due to the "protein corona" effect 194. Therefore, the use of targeting molecules to functionalize NP-mRNA should be carefully considered.

Finally, most LNP-mRNA therapies for cancer patients are still in the early stages of clinical trials. Positive therapeutic results from these clinical studies will support promising candidate drugs' progression to the next evaluation stage. More importantly, studying clinical data will provide valuable insights into optimizing suitable mRNA delivery methods, thereby promoting the translation of mRNA nanotechnology into clinical research. In conclusion, driving the development of mRNA delivery technology in tumor immunotherapy requires more effort. We look forward to continuous innovation and optimization of mRNA technology, bringing novel and effective results to life sciences and medical research.

## Figures and Tables

**Table 1:** The application of mRNA

Application	Agent	Disease/Condition	Reference
Protein replacement therapy	PTEN mRNA	subcutaneous PTEN-mutated melanoma and orthotopic PTEN -null prostate tumor models	6
	KDM6A mRNA	bladder cancer	7
	*TP53* mRNA	hepatocellular carcinoma and non-small-cell lung cancer	8
Gene editing	Cas9 mRNA	Lung-, spleen- and liver-targeted selective organ targeting (SORT) lipid nanoparticles	9
	mRNA/single-guide RNA	tissue-selective mRNA delivery and CRISPR-Cas9 gene editing in spleen, liver and lungs.	10

**Table 2:** The application of mRNA in cancer immunotherapy

	Agent	Disease/Condition	Reference
Cancer vaccines	luciferase and carcinoembryonic antigen (CEA) mRNA	colon carcinoma	20
	gp100 mRNA	melanoma	21
	prostate-specific antigen (PSA) mRNA to DC	prostate cancer	22
	mRNA coding for the melanoma associated antigens	melanoma	24
	tumor-associated antigens (TAAs) mRNA	triple negative breast cancer	NCT02316457
	personalized mRNA vaccines	melanoma	25
Adoptive T-cell therapy	chimeric antigen receptor (CAR) mRNA to T cells	acute lymphoblastic leukemia	28
	c-Met-CAR mRNA	metastatic breast cancer	29
mRNA-encoded antibodies	BiTE encoding mRNA with 1-methylpseudouridine (RiboMAB) simultaneously targets CD3 and one of three tumor-associated antigens (TAAs)	ovarian cancer, gastric adenocarcinoma	26
	mRNA encoding bispecifically binds and neutralizes CCL2 and CCL5 (BisCCL2/5i)	primary liver cancer, liver metastasis of colorectal and pancreatic cancers	30
mRNA-encoded immunomodulatory proteins	mRNA encoding four tumor-regressing cytokines (IL-12 single chain, IFN-α, GM-CSF, and IL-15)	colorectal cancer, melanoma	27
	mRNAs encoding OX40L, IL-36γ, and IL-23	hepatoma, colon carcinoma	31

**Table 3:** The pros and cons of each mRNA NPs

Nanoparticles carrier	Pros	Cons
Liposomes	Good biocompatibility; Multifunctionality; Can encapsulate hydrophilic and hydrophobic drugs at the same time.	Complex preparation process; Less amenable to scalability
Lipid nanoparticles	Encapsulation of mRNA using lipid bilayers prevents enzymatic degradation in the somatic circulation; Simple chemical synthesis of lipid-related components; Robust encapsulation capabilities.	The reticuloendothelial system (RES) or multiple organs can remove LNP from somatic circulation limiting its effectiveness.
Polymer nanoparticles	Forms stable complexes with RNA through electrostatic interactions, thus resisting degradation and promoting cellular uptake; Highly modifiable (easily functionalized, optimized drug release kinetics); Robust nucleic acid loading capacity.	Have the cytotoxicity; High molecular weight polymers are prone to aggregation *in vivo*.
Hybrid nanoparticles	Diverse structures; Better stability and biocompatibility.	Complexity of design and synthesis; Poor biodegradability; High production cost.
Protein nanoparticles	Good biocompatibility, adjustability and biodegradability	Low encapsulation efficiency; Endotoxin-induced toxicity; Abrupt drug release.
Exosomes	Good biocompatibility	Complexity of extraction
Peptide-based nanovesicles	High drug loading capacity; Good biocompatibility; Strong customizability	Poor stability; High production costs; Prone to immune reactions
OMVs	Strong immunogenicity; Multifunctional	High production costs; Poor stability; Unclear mechanisms

**Table 4:** mRNA NPs targeting different immune cells.

Immune cells	Animal models	Administration routes	Injection routes	Dosing amount	Dosing times	Results	Reference
DCs	TC-1 subcutaneous tumor model	Day 5, 10, 15	Intravenous	10 μg mRNA	Three	Systemic LPR treatment improved the median survival time of TC-1-inoculated mice and was even superior in controlling tumor growth.	64
	TC-1, B16F0, EG7-OVA subcutaneous tumor model	Day 7, 9	Intradermal	56 μl of LPR	Two	MART1 and OVA triMN-LPR triggered a significant delay in the B16F0 and EG7 tumor growth.	65
	B16-OVA, B16 subcutaneous tumor model	Day 4, 8, 12	Subcutaneous	10 μg mRNA	Three	C1 mRNA vaccine with a self-antigen and model antigen inhibited the growth of tumors.	66
	/	Twice of two week	Intranasal	10 μg mRNA	Two	CP 2k/mRNA induced significantly higher titers of IgG1 and IgG2a than naked mRNA.	67
	LL2 orthotopic tumor model	Day 4, 11, 18	Subcutaneous	10 μg mRNA	Three	DOTAP/DP7-C/neoantigen mRNA complexes exert a better antitumor effect	68
T cells	E0771 tumor model		Intravenous	mCherry mRNA (0.6 mg) .	One	aCD3-LNPs transfected 2-7% of circulating T cells and 2-4% of splenic T cells respectively.	69
	Ai6 mice carrying a Cre reporter allele	Every 24 h	Intravenous	10 μg Cre mRNA	Three or five	The sequential administrations of the targeted mRNA-LNPs resulted in increasing Cre-induced genetic recombination with increased number of injections in both the spleen and lymph nodes.	70
	A mouse model of heart failure	/	Intravenous	10 μg LNP	One	Marked functional improvements were observed in injured mice.	71
	LNCaP C42 orthotopic tumor model	Day 0, 7, 14, 21, 28, 35	Intravenous	50 μg mRNA	Six	CAR-encoding or TCR-encoding mRNA particles can genetically reprogram circulating T cells to induce antitumor responses.	72
	A20, CT26, or B16F10 subcutaneous tumor model	Day 5, 7, 9, 11, 13, 15	Intratumoral	10 μg mRNA	Six	PL1 nanoparticles delivering the costimulatory OX40 mRNA could enhance the immunotherapeutic effects of anti-OX40 Ab therapy in different mouse models.	73
NK cells	Orthotopic HCC tumor model	Day 22, 29	Intravenous	2×10 ^6^ cells	Two	CAR-DLNP mNK cell therapy decreased tumor proliferation and increased tumor cell apoptosis.	74
Macrophages	Gliomas tumor model	3 doses/week for 3 weeks	Retro-orbital	30 µg mRNA	Nine	Nanoparticles can deliver genes encoding master regulators of macrophage polarization to re-program immunosuppressive macrophages into tumor-clearing phenotypes.	75
	B16F10 tumor model	/	Intravenous	0.6 mg/kg mRNA	One	CD11b^hi^ macrophage-tropism of Lipid 16 would increase the mRNA delivery to a solid tumor.	76
B cells	C57BL/6 mice	/	Intravenous	0.75 ~ 2.25 mg/kg mRNA	One	Approximately 60 pg of luciferase protein was produced per million B cells at the highest OF-Deg-Lin LNP dose.	77
	B16F10 tumor model	Every other day	Intratumoral	2 μg IL-12 mRNA or 6 μg IL-27 mRNA	Six	Intratumoral administration of IL-12 and IL-27 mRNAs by DAL-LNP promoted sustained inhibition of B16F10 melanoma growth without causing significant toxicity.	78
Neutrophils	MC38 tumor model	/	Intratumoral	5 μg of OX40L mRNA	One	The three most abundant myeloid cell types within tumors are macrophages, monocytes, and granulocytes; all expressed OX40L above control mRNA-dosed tumors.	31
	B16F10 tumor model	Days 3, 6,10	Subcutaneous	10 μg mRNA	Three	Treatment of B16F10 melanoma tumors with lipid nanoparticles containing mRNA coding for the tumor-associated antigens gp100 and TRP2 resulted in tumor shrinkage and extended the overall survival of the treated mice.	79

**Table 5:** Current clinical trials of mRNA-nanoparticle therapy against cancer.

Cancer type	mRNA	Nanoparticle carrier	Phase	NCT number	Reference
Metastatic non-small cell lung cancer	BI 1361849	LNPs	I/II	NCT03164772	https://clinicaltrials.gov/ct2/show/NCT03164772
Malignant solid tumors	BNT113	Liposomes	II	NCT04534205	https://clinicaltrials.gov/ct2/show/NCT04534205
Squamous cell carcinoma, head and neck neoplasm, cervical neoplasm, penile neoplasms malignant	HARE-40	LNPs	I/II	NCT03418480	https://clinicaltrials.gov/ct2/show/NCT03418480
Melanoma	Lipo-MERIT	LNPs	I	NCT02410733	https://clinicaltrials.gov/ct2/show/NCT02410733
Melanoma, colon cancer, gastrointestinal cancer, genitourinary cancer, hepatocellular cancer	mRNA-4650	LNPs	I/II	NCT03480152	https://clinicaltrials.gov/ct2/show/NCT03480152
Non-small cell lung cancer, pancreatic neoplasms, colorectal neoplasms	mRNA-5671/V941	LNPs	I	NCT03948763	https://clinicaltrials.gov/ct2/show/NCT03948763
Adult glioblastoma	RNA-LPs	Liposomes	I	NCT04573140	https://clinicaltrials.gov/ct2/show/NCT04573140
Melanoma/colorectal cancer	RO7198457	LNPs	II	NCT03815058	https://clinicaltrials.gov/ct2/show/NCT03815058
	BNT122			NCT04486378	
Metastatic neoplasm	SAR441000	LNPs	I	NCT03871348	https://clinicaltrials.gov/ct2/show/NCT03871348
Triple negative breast cancer	TNBC-MERIT	Liposomes	I	NCT02316457	https://clinicaltrials.gov/ct2/show/NCT02316457
Ovarian cancer	W_ova1	Liposomes	I	NCT04163094	https://clinicaltrials.gov/ct2/show/NCT04163094
Non-small cell lung cancer	CV9202	Protamine	I/II	NCT03164772	https://clinicaltrials.gov/ct2/show/NCT03164772
Melanoma/non-small cell lung cancer	mRNA-4157	LNPs	I/II/III	NCT03897881, NCT03313778	https://clinicaltrials.gov/ct2/show/NCT03313778
